# Acinar Cell Apoptosis in *Serpini2*-Deficient Mice Models Pancreatic Insufficiency

**DOI:** 10.1371/journal.pgen.0010038

**Published:** 2005-09-23

**Authors:** Stacie K Loftus, Jennifer L Cannons, Arturo Incao, Evgenia Pak, Amy Chen, Patricia M Zerfas, Mark A Bryant, Leslie G Biesecker, Pamela L Schwartzberg, William J Pavan

**Affiliations:** 1 Genetic Disease Research Branch, National Human Genome Research Institute, National Institutes of Health, Bethesda, Maryland, United States of America; 2 Division of Veterinary Resources, Office of Research Services, National Institutes of Health, Bethesda, Maryland, United States of America; The Jackson Laboratory, United States of America

## Abstract

Pancreatic insufficiency (PI) when left untreated results in a state of malnutrition due to an inability to absorb nutrients. Frequently, PI is diagnosed as part of a larger clinical presentation in cystic fibrosis or Shwachman–Diamond syndrome. In this study, a mouse model for isolated exocrine PI was identified in a mouse line generated by a transgene insertion. The trait is inherited in an autosomal recessive pattern, and homozygous animals are growth retarded, have abnormal immunity, and have reduced life span. Mice with the disease locus, named *pequeño (pq),* exhibit progressive apoptosis of pancreatic acinar cells with severe exocrine acinar cell loss by 8 wk of age, while the islets and ductal tissue persist. The mutation in *pq/pq* mice results from a random transgene insertion. Molecular characterization of the transgene insertion site by fluorescent in situ hybridization and genomic deletion mapping identified an approximately 210-kb deletion on Chromosome 3, deleting two genes. One of these genes, *Serpini2,* encodes a protein that is a member of the serpin family of protease inhibitors. Reintroduction of only the *Serpini2* gene by bacterial artificial chromosome transgenic complementation corrected the acinar cell defect as well as body weight and immune phenotypes, showing that deletion of *Serpini2* causes the pequeño phenotype. Dietary supplementation of pancreatic enzymes also corrected body size, body weight, and immunodeficiency, and increased the life span of *Serpini2*-deficient mice, despite continued acinar cell loss. To our knowledge, this study describes the first characterized genetic animal model for isolated PI. Genetic complementation of the transgene insertion mutant demonstrates that *Serpini2* deficiency directly results in the acinar cell apoptosis, malabsorption, and malnutrition observed in *pq/pq* mice. The rescue of growth retardation, immunodeficiency, and mortality by either *Serpini2* bacterial artificial chromosome transgenic expression or by pancreatic enzyme supplementation demonstrates that these phenotypes are secondary to malnutrition in *pq/pq* mice.

## Synopsis

Pancreatic insufficiency is defined by the inability to digest and absorb nutrients due to the loss of pancreatic enzyme function or loss of the acinar cells that produce the enzymes. In this manuscript the authors have described a mouse model of pancreatic insufficiency characterized by the specific loss of pancreatic acinar cells. This specific acinar cell loss results in mice that are unable to digest and absorb nutrients from the diet, stunting the animal's growth and giving rise to immunological anomalies. The authors have identified a serendipitous transgene insertion/deletion encompassing the mouse *Serpini2* gene locus as the source of the phenotypes observed. Reintroduction of the *Serpini2* gene, a member of the serpin family of serine cysteine protease inhibitors, by bacterial artificial chromosome complementation corrects the pancreatic and immunological phenotypes of the disorder, confirming *Serpini2* as the responsible gene. Reintroduction of pancreatic enzymes through diet supplementation is also capable of correcting the reduction in size and weight, reduction in viability, and immunological deficiencies, indicating that these phenotypes are secondary to malnutrition alone. This work provides a new mouse model for investigation of malnutrition/malabsorption due to pancreatic insufficiency and identifies a novel function for the serpin family member *Serpini2*.

## Introduction

The ability to ectopically express novel genes in the mouse genome via introduction of transgenic sequences has proved to be a valuable tool in the study of mammalian development and disease. However, in approximately 5% of the transgenic lines produced, an insertional mutation at the genomic integration locus also occurs [1]. Often the insertion of the transgene is accompanied by a loss of surrounding genomic sequences and, dependent on the location of nearby coding and regulatory sequences, will produce an observable phenotype. We have been able to take advantage of such a transgene insertion/deletion event to identify and characterize a mouse model of pancreatic insufficiency (PI).

PI or a lack of pancreatic enzymes can lead to malabsorption due to an inability to digest and absorb nutrients. The most common genetic disorder that exhibits PI is cystic fibrosis (CF), as 95% of patients with CF have pancreatic acinar cell loss, with fatty cell replacement and interstitial fibrosis [2]. A second example of a syndrome that involves PI is the Shwachman–Diamond syndrome (SDS). Patients present with PI due to pancreatic acinar cell loss, but also have clinical manifestations of short stature, neutropenia, pancytopenia and predisposition to acute myelogenous leukemia [3].

Animal models of acute PI have been created by inducing pancreatic acinar cell apoptosis, either by administration of the synthetic cholecystokinin analog caerulein [4] or by physical blockage of the pancreas by ductal ligation [5]. However, defined genetic animal models of PI have been lacking. Only recently has a genetic model for an inbred CF mouse been described that recapitulates pancreas involvement of the disorder; however, this animal also manifests other features of CF [6].

In this paper we describe a new mouse model for exocrine PI that arose serendipitously in a line of mice carrying a transgene insertion. This insertion event resulted in a genomic deletion of the gene encoding the protease inhibitor SERPINI2/ PANCPIN. The mutant allele, termed *pequeño* (which is the Spanish word for “small”), is characterized by severe exocrine acinar cell loss at 8 wk of age, while islets and ductal tissue are spared. The disorder is inherited in an autosomal recessive pattern, and untreated homozygotes are malnourished, with a body weight one-third smaller than control littermates. Secondary to the malnutrition, these animals also have compromised immunity and a reduced life span. Administration of pancreatic enzyme diet supplementation is sufficient to reverse the effects of malnutrition in *pq/pq* animals, counteracting the growth defects, decreased viability, and immunodeficiency.

## Results

### Identification and Pathology of the Pequeño Mouse Line

**Figure 1:**
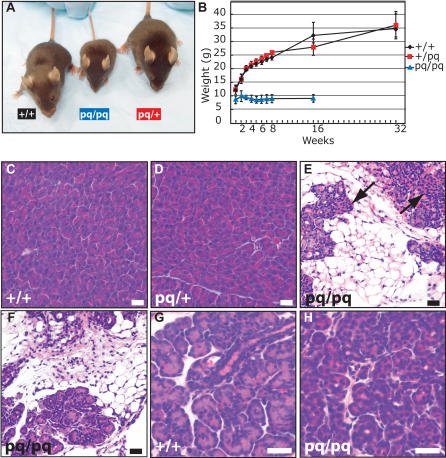
Acinar Cell Loss in *pequeño* Mice (A) Size of *pq/pq* mice in relation to wild-type and heterozygous littermates at 8 wk of age. (B) Average weight of *n =* 6 males for each genotype over a 32-wk period. (C–H) Pancreas hematoxylin and eosin histological analyses were performed on 8-wk-old (C, D, and E), 3-wk-old (F), and 1-wk-old (G and H) mice. Wild-type (*+,+*) acinar cells (C) and heterozygous (*pq/+*) acinar cells (D) at 8 wk of age were morphologically normal. This is in contrast to homozygous *(pq/pq)* animals at 8 wk of age (E) and 3 wk of age (F), where there is a severe loss of exocrine pancreatic acinar cells while duct cells and islet cells (arrow) are normal. Acinar cells are present in pancreas samples from 1-wk-old *pq/pq* mice (H), but the cells are smaller and show reduced cytosolic volume when compared to wild-type littermates (G). Bar represents 25 μm.

In offspring of a p3pTVA-B line of transgenic mice that were bred to homozygosity, we observed runted mice with a reduced viability phenotype that we called pequeño (Figure 1A). Line p3pTVA-B was one of five independent transgenic mouse lines generated using the *Pax3* promoter to drive expression of the avian retroviral receptor *tv-a* [7]. The four other lines generated did not produce the pequeño phenotype, suggesting that it was the site of transgene insertion that caused the phenotype. Analyses of 34 progeny resulting from the intercross of two obligate heterozygous mice demonstrated that 24 transgenic offspring (71%) were produced, in line with the expected Mendelian ratio. Six of the transgene-positive mice (6/34, 17.6%) were significantly smaller at weaning, suggesting that they were homozygous for a recessive mutation. Fluorescent in situ hybridization (FISH) analyses of metaphase spreads from three pequeño animals using the p3pTVA construct as a probe were performed. All three mice were homozygous for the transgene insertion on mouse Chromosome 3 (data not shown). We concluded that the pequeño phenotype is due to a homozygous mutation *(pq/pq)* that is linked to the transgene insertion and that the phenotype is inherited in an autosomal recessive pattern, as the heterozygotes had no apparent manifestations.

The *pq/pq* mice were on average one-third the weight of wild-type littermates (Figure 1B) and produced a gray-colored stool. Pathological assessment at 8 wk of age revealed that the spleen and thymus were dramatically reduced in size and the pancreas was abnormal by histological analyses of affected animals when compared to unaffected littermates. Gross skeletal abnormalities were not visible by X-ray or skeletal preparation (data not shown). The thymus of homozygous mutants (*n =* 6) were about 4-fold smaller than wild-type littermates (*n =* 6) (2.90% versus 11.44% thymus/brain weight; *p* < 2.5 × 10^−5^). Similar results were observed for the spleen (4.05% versus 16.7% spleen/brain weight; *p* < 9.7 *×* 10^−7^). Histological examination of the endocrine pancreas for *pq/pq* mice revealed that islets and ductal tissue appeared normal. Analysis of the exocrine pancreas showed a striking loss of acinar cells (Figure 1). Histological evaluation of the pancreas of younger animals at 1 wk of age showed abnormal acinar cells that contained fewer zymogen granules (ZGs) than those of littermate controls (Figure 1G and 1H). However, by 3 wk of age *pq/pq* animals had only about one-half the number of the pancreatic acinar cells when compared to wild-type, despite the normal appearance of ductal and endocrine islet cells (Figure 1F). This observation indicated that extensive acinar cell loss was occurring between 1 and 3 wk of age in *pq/pq* animals.

**Figure 2:**
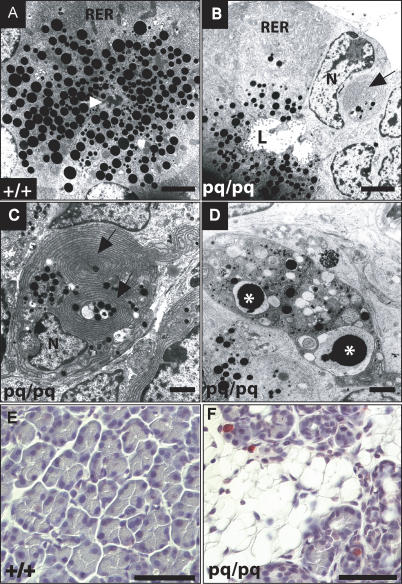
Electron Microscopy Shows *pequeño* Acinar Cells Are Present but Undergoing Apoptosis at 1 Wk of Age (A) Wild-type acinar cells have normal cellular morphology and contained abundant numbers of ZGs (seen as black circular vesicles containing electron-dense material), and electron-dense material from ZGs is present in the ductal lumen (white arrow). (B–D) *pq/pq* mice have fewer and smaller ZGs and no dense material in the ductal lumen (L) when compared to wild-type animals. A significant number of acinar cells are undergoing apoptosis, as indicated by the presence of cellular phagocytosis (C and D), rough endoplasmic reticulum whirling bodies (B and C) (black arrows), and nuclear condensation and fragmentation (D) (asterisk). (E and F) TUNEL staining of 3-wk-old pancreas tissue from wild-type (E) and *pq/pq* (F) mice indicates an increase in TUNEL-positive, red-fushcin-staining cells in *pq/pq* mice as compared to wild-type. L, lumen; N, nucleus; RER, rough endoplasmic reticulum. For (A–D) bar represents 5 μm. For (E and F) bar represents 50 μm.

Further analyses of the pancreas from 1-wk-old *pq/pq* animals by electron microscopy revealed a reduction in size and number of dense ZGs in the cytoplasm of the *pq/pq* acinar cells and a lack of dense material within the ductal network (Figure 2). In contrast, the pancreas of wild-type littermates contained many ZGs, and electron-dense material representing large quantities of enzymes is found in the pancreatic ducts (Figure 2A). Additionally, many *pq/pq* acinar cells were found to exhibit hallmark features of cells undergoing apoptosis. These acinar cells were smaller than adjacent cells and contained compact cytoplasmic organelles, condensed nuclear chromatin, and concentrically whirled bodies of rough endoplasmic reticulum (Figure 2B–2D). In addition, these acinar cells were observed being phagocytosed by neighboring epithelial cells while some were sloughed directly into the ductal lumina (Figure 2B–2D) [8]. As a second independent method of assessing apoptosis, pancreatic tissue was analyzed for DNA fragmentation using terminal deoxynucleotidyl transferase biotin–dUTP nick end labeling (TUNEL) staining. Pancreas sections from two animals in each group (wild-type and *pq/pq*) were counted for TUNEL staining in 12 independent, randomly selected fields (Figure 2E and 2F). The number of TUNEL-positive cells for *pq/pq* animals (mean counts of 7.2 and 4.9 cells per field) was significantly increased when compared to that of the wild-type (0.33 and 0.25 cells per field). Analyses of the four pair-wise comparisons were statistically significant (*p* < 0.01). Taken together, these results are consistent with apoptosis as the major cause of acinar cell loss.

**Figure 3:**
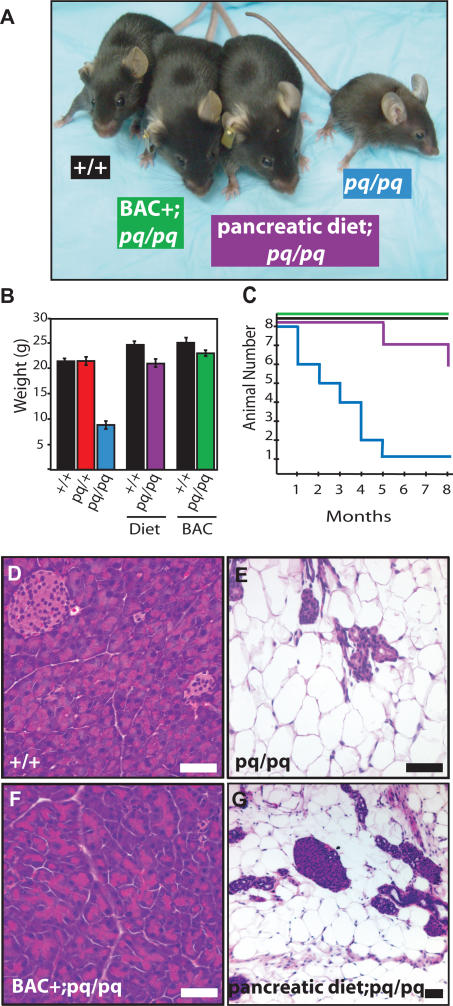
*pequeño* Malnutrition Is Rescued by BAC Transgenic Expressing *Serpini2* Gene or Pancreatic Enzyme Diet Supplementation (A–C) Treatment of *pq/pq* mice (blue) pharmacologically with daily pancreatic enzyme diet supplement (purple) or genetically with BAC 150E1 transgene (green), rescues (A) size, (B) weight, and (C) animal viability in comparison to wild-type mice (black). For (B) weight measurements are averaged from *n =* 6 males from each genotype. (D–G) Hematoxylin and eosin staining of pancreas sections from 8-wk-old mice. Wild-type mice (D) have normal pancreatic acinar cells present and normal pancreatic morphology while in (E) *pq/pq* mice there is a loss of acinar cells. In the BAC+; *pq/pq* mice (F), pancreas acinar cells are present. The acinar cell loss in *pq/pq* mice treated with enzyme supplementation (G) is the same as untreated *pq/pq* mice (A–C), although diet supplementation is effective in combating the effects of malnutrition. Bar represents 25 μm.

To test the hypothesis that growth failure is caused by PI, *pq/pq* animals were treated with a dietary supplementation of pancreatic enzymes in addition to a normal diet and assessed for correction of the malnutrition (Figure 3). Animals supplemented with a pancreatic enzyme diet were monitored for size, weight, and viability. Treated *pq/pq* mice gained both weight and length when compared with untreated *pq/pq* animals (Figure 3A and 3B). As expected, the enzyme supplement did not correct the acinar cell deficiency (Figure 3G). We conclude that the PI alone was responsible for the growth retardation in *pq/pq* mice (Figure 3).

### Identification of the Gene Responsible for the Pequeño Phenotype

**Figure 4:**
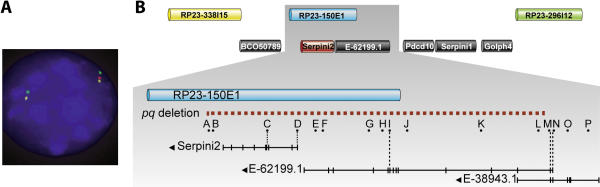
Pequeño Phenotype Is the Result of a 210-kb Genomic Deletion (A) FISH to interphase *pq/+* chromosome spreads with a p3pTVA transgene probe (red) locates the transgene insertion on mouse Chromosome 3 between BACs RP23-338I15 (yellow) and RP23-296I12 (green). (B) The genomic region defined by BAC FISH localization contained six known or predicted genes, one of which, *Serpini2,* is highly expressed in pancreas and adipose tissue. PCR primer sets A–P (Table S1) were used to characterize and define the identified ~210-kb deletion, indicated by red dashed line. PCR primer sets that correspond to known and predicted exon sequences are indicated by black vertical dashed lines. The genomic deletion encompassed the entire *Serpini2* gene locus and exons 4 to 17 of the predicted gene E-62199.1/E-38943.1.

To refine the location of the transgene insertion site in pequeño mice, the p3pTVA transgene was hybridized to heterozygous *pq/+* chromosome spreads in combination with bacterial artificial chromosomes (BACs) spanning Chromosome 3 using multicolor FISH. BACs spanning 52,290,000–85,000,000 bp of Chromosome 3 were identified with NCBI Map Viewer (http://www.ncbi.nlm.nih.gov/mapview/) mouse build 31, and obtained from Children's Hospital Oakland Research Institute (http://www.chori.org/bacpac). This analysis showed that the BAC clones RP23-388I15 and RP23-296I12 flanked the p3pTVA transgene insertion and mapped the insertion to a 2.3-MB region (Figure 4). This region contained four genes of known function and several putative genes represented by expressed sequence tags and/or gene prediction algorithms (Figure 4B). One candidate gene in the region, *Serpini2,* a serpin protein family member, is predicted to be a serine-cysteine protease inhibitor. In addition, *Serpini2* has been shown to be expressed at high levels in pancreas and adipose tissue [9,10], making it an excellent candidate gene for the pequeño phenotype.

The genomic draft sequence from the mouse February 2003 freeze build at the University of California at Santa Cruz (http://genome.ucsc.edu/cgi-bin/hgGateway) was used to design primers within the *Serpini2* gene and to the flanking genomic DNA (Figure 4B; Table S1). PCR analysis established that the transgene insertion caused in an approximately 210-kb genomic deletion at the locus, which included the entire *Serpini2* gene and continued distally to include several 3′ exons of Ensembl-predicted novel gene transcript sequences ENSMUST00000062199.1 (E-62199.1) and ENSMUST00000038943.1 (E-38943.1) (Figure 4B). The size and location of the deletion established the possibility that the pequeño phenotype could be a recessively inherited contiguous gene syndrome.

We reasoned that the restricted expression of the *Serpini2* gene in pancreas and adipose tissue as well as its proposed function as a serine protease inhibitor suggested that deletion of the *Serpini2* locus alone caused the pequeño phenotype. An alternative explanation was that deletion of the two Ensemble-predicted transcript sequences E-62199.1 and E-38943.1 contributed to the phenotype. Sequence analysis of the predicted exons for E-62199.1/E-38943.1 suggested they are a single gene and an ortholog of the human locus Unigene cluster Hs.376728. The human gene sequence and predicted mouse gene sequences are identical in exon–intron structure and are 75% identical at the amino acid level. The *pequeño* deletion includes the entire *Serpini2* gene locus and exons 14 to 17 of E-62199.1/E-38943.1, leaving the first three exons intact.

### BAC Transgene Complementation Cross Rescues Pequeño Acinar Cell Defect

In order to dissect the role of *Serpini2* and rule out the predicted genes as candidates for pequeño, we produced a BAC transgenic line, 150E1, to perform *Serpini2* complementation crosses with pequeño mice. BAC RP23-150E1 was selected using the University of California at Santa Cruz February 2003 browser because it contained the entire intact *Serpini2* gene and 71 kb of the upstream promoter region, but not a functional E-62199.1/E-38943.1 gene. BAC-positive (BAC+) transgenic animals were crossed with *pq/+* mice and the resulting double heterozygous BAC*+*; *pq/+* animals were backcrossed to *pq/+* animals. Animals were genotyped with primers to the p3pTVA transgene, a deletion-specific primer “77kb” (within the genomic deletion, but outside of BAC 150E1), and BAC vector end-specific primers. Transgenic BAC*+*; *pq/pq* animals were indistinguishable from BAC*−*; *+/+* and BAC*+*; *+/+* animals by size, weight and viability (see Figure 3A–3C). RT-PCR from pancreas RNA obtained from wild-type, BAC*−*; *pq/pq* null, and BAC*+*; *pq/pq* BAC-complemented animals was performed. The results demonstrated that *Serpini2* is not expressed in BAC*−*; *pq/pq* null animals and that the BAC transgene in BAC*+*; *pq/pq* animals restored expression of *Serpini2* in pancreas (data not shown). Histological analysis of BAC+; *pq/pq* animals revealed they had normal acinar cells, showing that *Serpini2* expression alone corrected the acinar cell defect phenotype (Figure 3D–3F). We conclude that deletion of the *Serpini2* gene, and not E-62199.1/E-38943.1, causes the pequeño phenotype of acinar cell loss leading to PI and subsequent growth retardation due to malabsorption and malnutrition.

### Immune Cell Defects in *pq/pq* Mice

The *pq/pq* mice without pancreatic diet supplementation exhibited decreased life span (typically 2–7 mo compared to over 1 y for normal mice). Interestingly, when *pq/pq* mice without pancreatic diet supplementation were housed in sterile cages with acidified water, their condition improved and their life span increased compared to *pq/pq* animals housed in standard conditions. This suggested that the *pq/pq* mice had compromised immune function and infections were contributing to the morbidity (data not shown). We therefore sought to characterize the immune system of the *pq/pq* mice.

**Figure 5:**
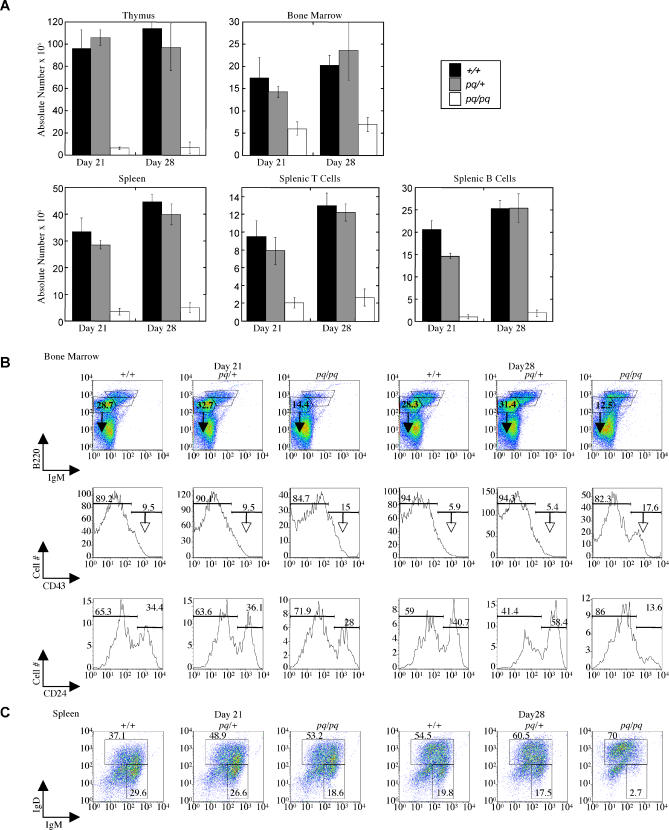
Decreased Lymphocyte Numbers and Impaired B cell Development in *pequeño* Mice (A) *pequeño* mice exhibit a striking reduction in the cellularity of the thymus, spleen and bone marrow at days 21 and 28 of life (*n =* 3). (B) *pequeño* mice show decreased early B cell progenitors in the bone marrow. Expression profiles of B220 and IgM on total lymphoid cells in bone marrow are shown for wild-type (top row, percentage of B220^+^IgM^−^ pro-/pre-B cell fraction are indicated). The B220^+^IgM^−^ pro-/pre-B cell fraction (filled arrow) was gated to analyze the developmental stages using CD43 (middle row). The B220^+^IgM^−^CD43^+^ (less mature) fraction (open arrow) was then gated on to analyze CD24 expression (bottom row). *pq/pq* mice exhibit an increase in the proportion of CD43^+^CD24^−^ B cells, indicating a block in the pre-/pro-B to early pro-B transition. (C) Flow cytometric analysis of surface IgM^+^/IgD^−^ expression on B220^+^ cells in the spleen indicates a decreased percentage of immature (IgM^+^/IgD^−^) B cells in the *pequeño* mice.

**Figure 6:**
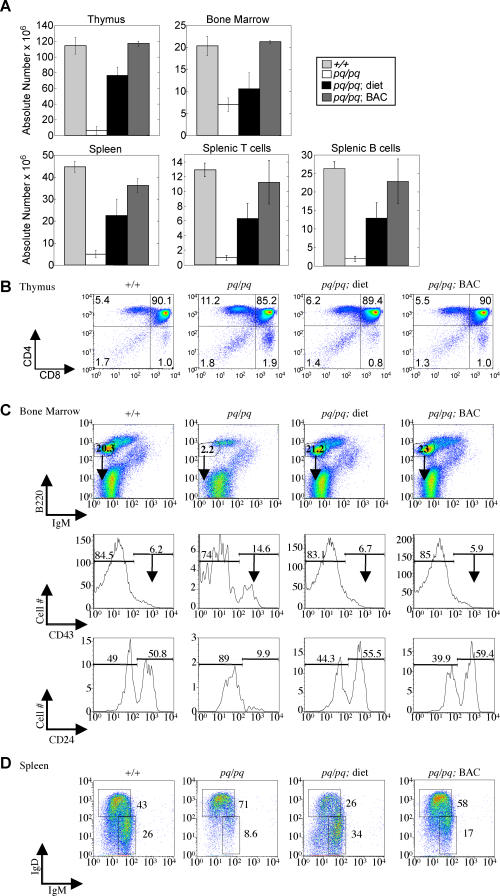
Pancreatic Enzyme Supplementation and BAC Transgenesis Rescue Lymphocyte Cells Numbers and Developmental Defects in *pequeño* Mice (A) *pequeño* mice with either enzyme supplementation (*pq/pq;* diet) or a BAC transgenic (*pq/pq;* BAC) display increased cellularity of the thymus, spleen, and bone marrow at day 28 of life (*n =* 4). (B–D) Pancreatic enzyme supplementation and the BAC transgenic rescue the developmental defects in the *pequeño* mice. (B) CD4 and CD8 expression profiles of the thymus. (C) Expression profiles of B220 and IgM on total lymphoid cells in bone marrow (top row). The B220^+^IgM^−^ pro-/pre-B cell fraction (filled arrow) was gated to analyze the developmental stages using CD43 (middle row). The B220^+^IgM^−^CD43^+^ (less mature) fraction (open arrow) was then gated on to analyze CD24 expression (bottom row). (D) Splenic expression profile analysis of surface IgM/IgD expression on B220^+^ cells .

Analyses of lymphoid organs revealed that *pq/pq* mice had a reduction in thymic, splenic, and bone marrow cellularity by 21 and 28 d after birth (Figure 5). In particular, spleens from *pq/pq* mice at 3–4 wk of age showed a dramatic reduction in the number of lymphocytes present, with T cell numbers reduced approximately 5-fold and B cell numbers reduced more than 10-fold. Thymocyte numbers were also severely reduced (10- to 20-fold), suggesting that the decrease in mature T cells resulted from reduced thymic output. Despite the dramatic reduction in thymic size, *pq/pq* mice had a normal thymocyte profile, with cells progressing from the most immature double negative (CD4^−^8^−^) population to double positive CD4^+^8^+^ cells and both CD4 and CD8 single-positive mature cells, with no clear block at any stage (Figure 6). In particular, the increase in the percentage of single-positive CD4^+^ and CD8^+^ cells argues that the cells exhibit no defects in selection and can progress to the most mature stages of thymic development. These results suggested that thymic development was not blocked per se, but that there was reduced cell input or viability at multiple stages.

We also observed a defect in B cell development. B lymphocytes develop in the bone marrow through distinct stages characterized by differential expression of cell surface markers and the ordered rearrangement of Ig heavy and light chain gene segments [11,12]. Wild-type mice have populations of B220^+^sIgM^−^ pro- and pre-B cells in their bone marrow that upregulate and express increasing levels of sIgM on their cell surfaces as they develop into immature and mature B cells. In contrast, *pq/pq* mice had fewer pro- and pre-B cells (B220^+^IgM^−^), and expression of IgM on immature cells was lower, particularly at day 28 (see Figure 5B). Within the B220^+^sIgM^−^ fractions, the most immature pro-B cell fractions, fractions A–C, are further defined by expression of CD43 (B220^+^IgM^−^CD43^+^) and differing expression of CD24 [13]. Fraction A is CD24^−^ and contains cells that first appear to be committed to the B cell lineage (pre-pro-B cells). Fractions B (early pro-B cells) and C (late pro-B cells) now express CD24 and initiate rearrangement at the IgH locus (B220^+^IgM^−^CD43^+^CD24^+^). Compared to wild-type, *pq/pq* pre-B cells manifested a higher percentage of B220^+^IgM^−^CD43^+^ cells, indicative of a partial block in B cell development prior to the pre-B cell stage. This block was more apparent as the mice aged (compare day 28 to day 21, Figure 5B). Moreover, within the CD43^+^ fraction, *pq/pq* pro-B cells expressed lower levels of CD24, demonstrating that this block occurred between the pre-proB and early pro-B cell stage. Similarly, in the spleen, reduced numbers of the most immature population of B cells (IgM^+^IgD^−^) were observed (Figure 5C), arguing that there was reduced production of B cells in the bone marrow.

### Pancreatic Diet Supplementation and BAC Transgene Rescues Immune System Defects

To determine whether the PI and subsequent malnourished state alone were contributing to the impaired immune system or whether there was an intrinsic defect in *pq/pq* lymphocytes due to the lack of SERPINI2, we sought to determine how the *pq/pq* mice would respond to dietetic supplementation with pancreatic enzymes in contrast to *BAC+*; *pq/pq* and *pq/pq* untreated animals. The pancreatic enzyme replacement therapy restored immune cell numbers in the *pq/pq* mice, suggesting immune system defects were secondary to malnutrition. Similarly, BAC+; *pq/pq* transgenic mice were also able to complement immune system defects. At 4 wk of age, enzyme supplementation resulted in a partial rescue, while animals with the BAC transgene were indistinguishable from wild-type animals with respect to cellularity of the spleen, thymus, and bone marrow and the numbers of mature T and B cells within the spleen (see Figure 6A). Moreover, both the diet supplementation and the BAC transgene insertion complemented bone marrow differentiation defects. *pq/pq* mice with either pancreatic enzyme diet supplementation or the BAC transgene had an increased B220^+^IgM^−^ fraction and normalized development of IgM^+^ immature B cells and the percentage of early progenitor B cells, as defined by CD43 and CD24, when compared to *pq/pq* animals without either enzyme supplementation or BAC transgene introduction (Figure 6C). Splenic B cell profiles were also normalized, with increased percentages of early newly emigrated B cells (Figure 6D) in enzyme-treated and BAC transgenic *pq/pq* animals. Thus, the immune defects in *pq/pq* mice appear to be the in large part secondary to pancreatic exocrine deficiency, which leads to malnourishment caused by malabsorption.

## Discussion

We have identified a mutant with PI caused by a deletion of the protease inhibitor *Serpini2*. This phenotype (pequeño) is inherited in an autosomal recessive pattern and is to our knowledge the first published mouse mutant to genetically model PI. The mutation, caused by a transgene insertion, led to an approximately 210-kb genomic deletion that completely removes the *Serpini2* gene and a portion of the predicted gene E-62199.1/E-38943.1. Reintroduction of the *Serpini2* gene alone by BAC complementation corrected the acinar cell defect and improved immune function, demonstrating that deletion of the *Serpini2* gene is the primary genetic cause of the pequeño mouse phenotype.

*Serpini2* was initially identified and implicated as a potential tumor suppressor gene because it was downregulated in pancreatic cancer cell lines when compared to normal pancreas [9]. *Serpini2* is also known as *MEPI,* a gene expressed in normal breast myoepithelial cells, but not in malignant breast carcinoma cells [14]. We did not observe any overt phenotype in the *pequeño* mice with regard to mammary gland development/function. Female *pq/pq* mice that had been maintained on a pancreatic enzyme diet supplementation were able to rear litters of 8–10 pups with no difficulty. It is possible that loss of expression in mammary tissue does not affect mice mammary gland function. Alternatively, there is the potential that another serpin family member may be compensating for the loss of *Serpini2* activity, as has previously been observed for loss of *Serpinb6* function resulting in upregulation of *Serpinb1* expression [15].

SERPINI2 is a member of the serpin superfamily of proteins that have been implicated in a variety of functions including blood coagulation, angiogenesis, inflammation, and programmed cell death [16]. In humans, 33 serpins have been identified [17]. These proteins are further subdivided into two classes, inhibitory and non-inhibitory. The former group, which includes SERPINI2, is characterized by conformational structure changes that allow for formation of covalent acyl-enzyme intermediates with the proteins' target protease, and thus have been termed “suicide substrates” [18]. The target substrates for SERPINI2 are unknown.

Further insight into the cellular location and function of SERPINI2 has come from work with the rat homolog, ZG-46p. SERPINI2/ZG-46p has been characterized in the pancreas, located predominately in the Golgi and in ZGs [19], and was isolated from ZG membranes [20]. ZGs in the acinar cells contain inactive proenzyme precursors for digestive enzymes including trypsin, amylase, chymotrypsin, and carboxypeptidase. Normal acinar cells produce large quantities of granules that are transported from the trans-Golgi network to the luminal side of the acinar cell and are then secreted to the ductal lumen upon hormonal and neural signaling mechanisms. The contents of the ZGs are transported via ducts to the duodenum (pp. 429–430 of [21]), where these enzymes are required for proper digestion and absorption of food.

Given the subcellular location of SERPINI2 in the Golgi and ZGs, in addition to the predicted gene function as a serine protease inhibitor, we hypothesize that SERPINI2 may play a role in regulating the level of inactive zymogen precursors. The importance of tight regulation of active protease levels by protease inhibitors in the exocrine pancreas is well documented. This regulation is demonstrated by considering mutations in two genes that are mutated in human hereditary pancreatitis: the cationic trypsinogen gene *PRSS1* and *serine protease inhibitor Kazal type 1 (SPINK1)*. For a small number of individuals with chronic PI, mutations in either gene have been shown to alter this balance and lead to autodigestion of tissue with inflammation [22,23]. However, genetic animal models for these two genes are lacking.

It is also important to note that differences in the severity of pancreatitis may be regulated by the type of cellular response to the inflammation, whether apoptosis or necrosis. In several models of PI, acinar cell apoptosis causes a less severe pancreatitis phenotype [24–26], in contrast to the more severe pancreatitis that has been documented with acinar cell necrosis [25,27]. It is interesting to speculate that other less severe alleles than the null allele present in the *pequeño* mouse model may not have severe apoptosis and may demonstrate a pancreatitis phenotype similar to that observed in patients with *SPINK1* mutations.

Alternatively, SERPINI2 may function to allow proper sorting and/or transport of ZGs from the Golgi to the ductal lumen of the acinar cell. This hypothesis is consistent with the observations made of the acinar cells of 1-wk-old *pq/pq* mice that have yet to undergo apoptosis. In these acinar cells there is a dramatic loss of ZGs present in the cell and the ZGs appear smaller in size than those in wild-type and heterozygous littermates. Further analysis is required to differentiate these potential modes of action for SERPINI2.

It is clear that malnutrition is the primary cause of the observed *pequeño* phenotypes. The inability of *pq/pq* mice to adequately digest and absorb food from a young age adversely affects growth and immune system function. A large body of evidence argues that malnutrition, stress-induced hormones, and corticosteroids can adversely affect immune cell numbers and survival [28]. In addition, leptin, a pleiotropic molecule that regulates food intake and metabolic endocrine functions, also plays a role in immune and inflammatory responses [29]. Although the defects we observed here do not completely correlate with those associated with increased steroid exposure in mice, most studies of steroid exposure have examined short-term glucocorticoid exposure [30]. However, over-expression of a glucocorticoid receptor in the thymus produced results similar to ours, with reduced cellularity observed at multiple stages of T cell development [31]. Similar long-term studies have not examined B cell numbers or function. While we cannot rule out a direct effect of SERPINI2 on lymphocytes, the improvement of immune function by pancreatic diet supplementation strongly suggests that these defects are secondary to malnutrition, which could result in increased stress hormone and steroid production, decreased leptin levels, and/or a lack of nutrients.

It is interesting that growth retardation, PI, and immunodeficiency are present in both the *pequeño* mouse and the human disorder SDS. The human disease is a rare and clinically heterogeneous disorder that manifests PI, short stature, lipidosis of the pancreas, neutropenia, pancytopenia, and predisposition to acute myelogenous leukemia. Although we have not observed neutropenia in the blood of *pq/pq* mice (Figure S1; Table S2), it may be difficult to compare secondary immune defects in mouse and human, as effects of malnutrition and stress hormones may differ between the species. Nonetheless, the combination of PI and immune defects in this mouse mutant is intriguing, given that several recent studies indicate SDS may exhibit genetic heterogeneity [32–34].

The most common mutation for SDS has been identified and is a gene conversion caused by recombination of the ubiquitously expressed *SBDS* gene with the pseudogene *SBDSP* located 5.8 Mb away on Chromosome 7 [32]. The SBDS protein is predicted to be an RNA-processing enzyme based upon sequence homology to a yeast ortholog and cluster analysis data from microarray experiments [32]. Given the overlap in phenotypes of the *pequeño* mice and genetic heterogeneity predicted for patients with SDS, *Serpini2* is a candidate gene for analysis in the subset of patient samples in which no mutation in the *SBDS* gene can be identified.

## Materials and Methods

### Pancreatic enzyme diet.

The pancrezyme diet was provided as a supplementation to normal mouse chow Rodent NIH-31 Auto18–4 (Ziegler, Gardeners, Pennsylvania, United States). The supplement (Pancrezyme, Daniels Pharmaceuticals, St. Petersburg, Florida, United States) was mixed with Transgenic Dough diet (Bio-Serv, Frenchtown, New Jersey, United States) at a ratio of 0.9 g supplement/50 g of transgenic dough. Five grams per animal of supplemented transgenic dough mixture was provided once every 24 h.

### FISH.

Metaphase spreads were performed with standard air-drying technique from blood collected from retro-orbital bleeds and mouse spleens [35]. DNA was labeled by nick translation technique, essentially as described by Pinkel et al. [36] and Lichter et al. [37] with Spectrum Orange (Vysis, Downers Grove, Illinois, United States). On each slide 200 ng of labeled probe was applied. Repeat sequences were blocked with 10× excess Cot1 DNA, 10 μl of a hybridization mixture containing the labeled DNA, 50% formamide, 2× SSC, and 10% dextran sulfate (pH 7) and were denatured at 75 °C for 10 min, then incubated at 37 °C for 30 min. Slides were incubated for at least 1 h at 37 °C in 2× SSC, then dehydrated with 2-min ethanol washes (70%, 80%, and 90%). Slide denaturation was performed in 70% formamide/2× SSC for 2 min, then dehydrated at −20 °C in 2-min ethanol washes (70%, 80%, 90%, and 100%). Post-hybridization washes were performed at 45 °C as follows: (1) 50% formamide and 2× SSC for 15 min, (2) 0.1× SSC for 10 min. Slides were counterstained with DAPI-Antifade (250 ng/ul BM, Vector, Burlingame, California, United States).

### Production of BAC transgenic mice.

P3pTVA-transgenic mice were generated as described [7]. For BAC complementation analysis, fertilized oocytes obtained from matings of C57BL/6J mice were used for pronuclear injection of RP23-150E1 BAC DNA. BAC DNA was purified by CsCl gradient isolation and not linearized prior to injection. The concentrations of BAC DNA used for microinjection were 0.5–0.75 μg/ul. A total of 69 pups were born, five of which were positive for the BAC by PCR.

### Histology and electron microscopy.

Tissues evaluated by hematoxylin and eosin staining and light microscopy were fixed in 10% neutral buffered formalin routinely processed, embedded into paraffin, sectioned at 5 μm thick, and stained with hematoxylin and eosin, performed by Histoserve (Rockville, Maryland, United States). Pancreas tissue evaluated by electron microscopy was fixed in 2.5% glutaraldehyde in 0.1 M cacodylate buffer (pH 7.4) overnight. The tissue was washed with cacodylate buffer and postfixed with 1% OsO_4_ for 2 h. Tissue was washed again with 0.1 M cacodylate buffer, serially dehydrated in ethanol and propylene oxide and embedded in Eponate 12 resin (Ted Pella, Redding, California, United States). Thin sections, approximately 80 nm, were obtained by utilizing the Leica Ultracut-UCT ultramicrotome (Leica, Deerfield, Illinois, United States) and placed onto 400 mesh copper grids and stained with saturated uranyl acetate in 50% methanol and then with lead citrate. The grids were viewed in the Philips 410 electron microscope (FEI, Hillsboro, Oregon, United States) at 80 kV and images were recorded on Kodak (Rochester, New York, United States) SO-163 film.

### TUNEL staining.

Pancreas tissue for TUNEL staining was fixed in 4% paraformaldehyde, sectioned at 5 μm thick, with TUNEL staining performed by Histoserve. Visualization of TUNEL-positive cells was done using New Fushcin dye. Each of the results for the two wild-type animals (mean counts of 0.33 and 0.25 per field) was compared to each of the two results for the *pq/pq* animals (mean counts of 7.2 and 4.9 per field) in a Bonferroni multiple-comparison test (pp. 255–262 of [38]). Because the distributions had significantly different standard deviations, the test was repeated with the non-parametric Kruskal-Wallis test followed by Dunn's post test [38]. All four pair-wise comparisons were statistically significant at *p* < 0.01.

### Cell preparation and flow cytometry.

Bone marrow cells were obtained by injecting PBS containing 5% FCS through surgically extracted femurs. Splenic cells and thymocytes were obtained by dissociation of the tissue with a plastic mesh and rubber stopper from a 3-ml syringe. All cells were treated with red blood cell lysis solution. For flow cytometric analysis, cells were blocked with normal mouse serum and Fc block for 10 min, then stained with FITC-, PE-, CyChrome-, or biotin-conjugated antibodies for 30 min. Biotin-conjugated antibodies were detected with a secondary stain: streptavidin-APC. Analysis was performed on a FACScan (Becton-Dickinson, Palo Alto, California, United States) using Flowjo software. The following monoclonal antibodies were obtained from BD PharMingen (San Diego, California, United States): CD3, CD4, CD8, B220, CD19, CD43, CD24, CD11b, GR-1, Ter119, DX5, and IgM. Anti-IgD was purchased from Southern Biotechnology (Birmingham, Alabama, United States).

### Genotyping.

PCR reactions used 1× Genamp PCR buffer, 2.5 mM MgCl_2_, 0.2 mM dNTPs, 0.2 nM each primer, and 1 unit Amplitaq (Applied Biosystems, Foster City, California, United States). PCR conditions were 5 min. denaturation at 93 °C followed by 35 cycles of 93 °C for 30 sec, 58 °C for 30 sec, and 72 °C for 30 sec. BAC primers designed to flanking arm sequences were 3199R (CGCTGCAAAACGCTGACGGAACAGTAG) and 3670R (CTCAGCGTATGGTTGTCGCCGGATGTAT), and 11016F (ATGGGCAAATATTATACGCAGGCGACAAG) and 11595R (CGTATTAGCGGCCGCAAATTTATTAGAGCA). Primers within the *pequeño* deletion, but outside of BAC sequences were 77kbF (CTTGCCTCATCAATGCTAGG) and 77kbR (GGATAAGTCTATCTGACTTC). Primers specific to transgene insertion were P3p39 (CTGGAGCCTGTGGACTTGGAT) and TVA Rout2 (CAGTGATCAGCATCCACATGC).

For primers used to characterize the *pequeño* genomic deletion, see Table S1.

## Supporting Information

Figure S1 Detection of Neutrophils in the Bone Marrow of Wild-Type and *pq/pq* Mice (A) Flow cytometric analysis was conducted by gating on granulocytes using forward versus side light scatter and GR-1and CD11b staining in the bone marrow. Increased percentage of neutrophils in the bone marrow of *pq/pq* mice is due to elevated percentage of GR-1lo CD11b^+^ immature neutrophils.
(696 KB PDF)

Table S1 Primers Sets Used to Define Genomic *pequeño* Deletion (63 KB DOC)

Table S2 *pequeño* Mice Show Increased Percentage of Neutrophils in Blood (54 KB PDF)

### Accession Numbers

The OMIM (http://www.ncbi.nlm.nih.gov/entrez/query.fcgi?db=OMIM) accession numbers for the genes and gene products discussed in this article are CF (602421) and SDS (260400).

The MGI (http://www.informatics.jax.org/) accession number for *Serpini2* is 1915181.

The Ensembl (http://www.ensembl.org) accession numbers for the genes discussed in this article are *PRSS1* (ENSG00000173636), *SPINK1* (ENSG00000164266), and E-62199.1/E-38943.1 (ENSMUSG00000058653).

The UniGene (http://www.ncbi.nlm.nih.gov/entrez/query.fcgi?db=unigene) accession number of the human ortholog of the mouse gene *Serpini2* discussed in this article is Hs.376728.

## Abbreviations

BAC: bacterial artificial chromosome
CF: cystic fibrosis
FISH: fluorescent in situ hybridization
PI: pancreatic insufficiency
*pq,*: *pequeño;*
SDS: Shwachman-Diamond Syndrome
TUNEL: terminal deoxynucleotidyl transferase biotin–dUTP nick end labeling
ZG: zymogen granule
