# Supplementary material for: Acinar Cell Apoptosis in Serpini2-Deficient Mice Models Pancreatic Insufficiency
Source: PLoS Genet. 2005 Sep 23;1(3):e38. doi: 10.1371/journal.pgen.0010038 (PMC1231717; doi:10.1371/journal.pgen.0010038)
Supplement: Supplementary file 1 [file pgen.0010038.sg001.pdf]

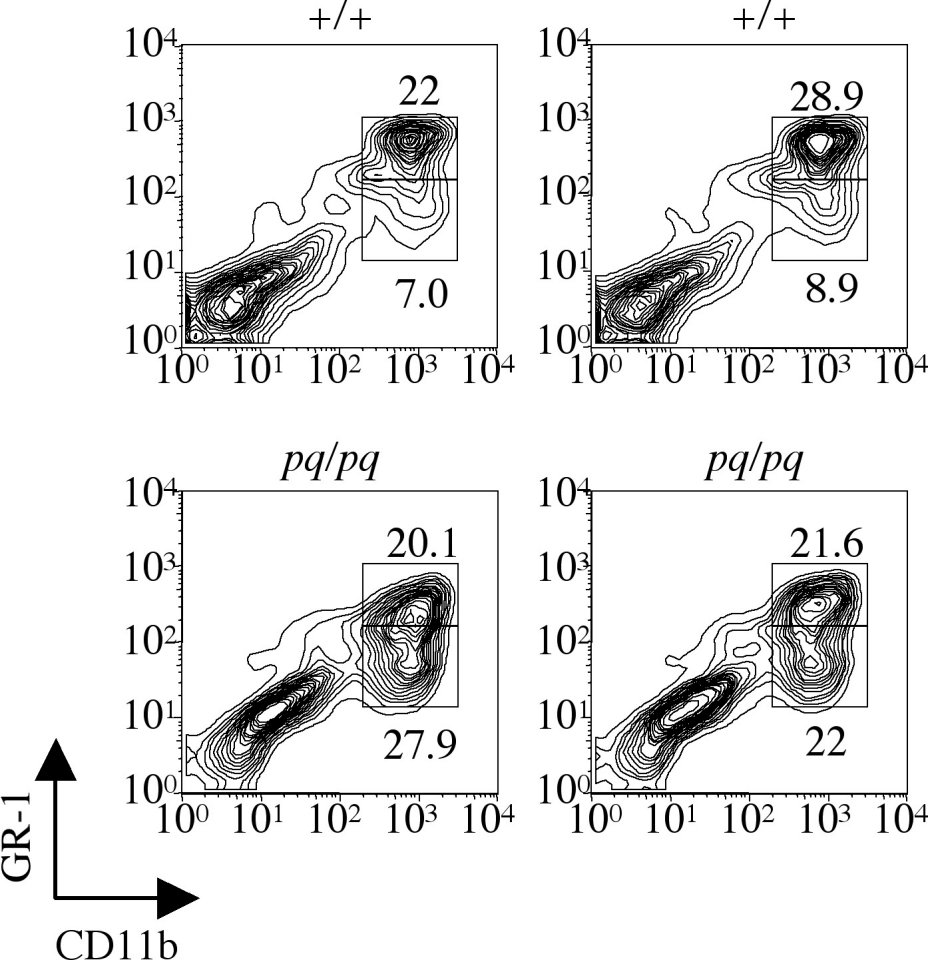

+/+
+/+
22
7.0
28.9
8.9
pq/pq
pq/pq
20.1
27.9
21.6
22
10^0
10^1
10^2
10^3
10^4
GR-1
CD11b
