# Supplementary material for: Acinar Cell Apoptosis in Serpini2-Deficient Mice Models Pancreatic Insufficiency
Source: PLoS Genet. 2005 Sep 23;1(3):e38. doi: 10.1371/journal.pgen.0010038 (PMC1231717; doi:10.1371/journal.pgen.0010038)
Supplement: Supplementary file 2 [file pgen.0010038.st002.pdf]

	pg/pg		*pg/pg*		+/+		+/+	
	Absolute (K/ml)	%	Absolute (K/ml)	%	Absolute (K/ml)	%	Absolute (K/ml)	%
WBC	3.74		2.78		5.16		4.76	
Neutrophils	1.39	37.18	1.10	39.39	0.61	11.75	0.51	10.64
Lymphocytes	1.96	52.46	1.47	52.99	4.47	86.55	4.15	87.19
Monocytes	0.22	5.76	0.20	7.32	0.07	1.31	0.02	0.46
Eosinophils	0.13	3.57	0.01	0.30	0.02	0.40	0.01	0.13
Basophils	0.04	1.02	0.00	0.00	0.00	0.00	0.00	0.00
