# Supplementary material for: Acinar Cell Apoptosis in Serpini2-Deficient Mice Models Pancreatic Insufficiency
Source: PLoS Genet. 2005 Sep 23;1(3):e38. doi: 10.1371/journal.pgen.0010038 (PMC1231717; doi:10.1371/journal.pgen.0010038)
Supplement: Supplementary file 3 [file pgen.0010038.st001.doc]

Supplementary Table S1.

Primers sets used to define genomic *pequeño* deletion

| **Figure 4 Primer set** | **Primer Name** | **Sequence** | **Within RP23-150E1** | **Within pequeno deletion** | **Product Size (bp)** |
| --- | --- | --- | --- | --- | --- |
| A | 3’(26kb)F | CACATGGCAAGCATGGCATTG | yes | no | 690 |
|  | 3’(26kb)R | CCTCTCAAAGGCTGCTGACTT |  |  |  |
|  |  |  |  |  |  |
| B | 3’(14kb)F | GCATTGCATGCCACATGCAG | yes | yes | 647 |
|  | 3’(14kb)R | CAAGTCTGTGAGATAAGCACAG |  |  |  |
|  |  |  |  |  |  |
| C | Spi14-2F | TACTAAGAAAGATGGCTCTACAG | yes | yes | 264 |
|  | Spi14-4R | CATGCAGCTCGGAGAACCATC |  |  |  |
|  |  |  |  |  |  |
| D | Spi14-int1R | AACTGTTCTGTAGCCTCTTG | yes | yes | 521 |
|  | Spi14-ex1F | GCTGACTTTGCAGTGGATC |  |  |  |
|  |  |  |  |  |  |
| E | 5’(10kb)F | GTGGCAAGTGATTACGAAGAG | yes | yes | 592 |
|  | 5’(10kb)R | GGATAGATCCTCATTGTGTTAC |  |  |  |
|  |  |  |  |  |  |
| F | 5’(17kb)F | GCTAATCTGGTGATGTCCTG | yes | yes | 515 |
|  | 5’(17kb)R | GAAGTCACTTACTCTACCTGAC |  |  |  |
|  |  |  |  |  |  |
| G | 5'(40kb)F | CTTCTCACACATGATTTCATG | yes | yes | 613 |
|  | 5'(40kb)R | GCCATTTGGAGATTATGCAG |  |  |  |
|  |  |  |  |  |  |
| H | 5'(52kb)F | GAACATAAGACAGTGTGAGG | yes | yes | 432 |
|  | 5'(52kb)R | CACCACTTACTATCATCTTAG |  |  |  |
|  |  |  |  |  |  |
| I | 5'(64kb)F | GGTAGAAGTGGTAGCAGATC | yes | yes | 630 |
|  | 5'(64kb)R | CTGGCTCATGTCTTCATATC |  |  |  |
|  |  |  |  |  |  |
| J | 5’(77kb)F | CTTGCCTCATCAATGCTAGG | no | yes | 650 |
|  | 5’(77kb)R | GGATAAGTCTATCTGACTTC |  |  |  |
|  |  |  |  |  |  |
| K | 5'(113kb)F | CCTTACTCTAGACTCTCTAC | no | yes | 497 |
|  | 5’(113kb)R | AGTGACACTTCTCAGTGCC |  |  |  |
|  |  |  |  |  |  |
| L | 5'(155kb)F | GCTGTAGACATAAGAAGTCAG | no | yes | 612 |
|  | 5’(155kb)R | GGTACCCTCCTTGAGTTAGTAC |  |  |  |
|  |  |  |  |  |  |
| M | 62199 Ex3 F | CCTGTATCTTCCATTCAACTG | no | no | 444 |
|  | 62199 Ex3 R | CTTAGCCAGTTTGCATGAAC |  |  |  |
|  |  |  |  |  |  |
| N | 62199Ex2F | GAGTGCTAAGACATGTCAGC | no | no | 379 |
|  | 62199Ex2R | GGCTGCTCAATTTGGTCTC |  |  |  |
|  |  |  |  |  |  |
| O | 5'(167kb)F | CGCTCCCTAAGGACCACAC | no | no | 447 |
|  | 5’(167kb)R | CAGGTGCAGTGGTATACAC |  |  |  |
|  |  |  |  |  |  |
| P | 5'(200kb)F | GACTTCTCACTGGTACAAG | no | no | 370 |
|  | 5’(200kb)R | AAGGCTCTGTGTTTCTGGAC |  |  |  |
|  |  |  |  |  |  |
| * | TvaR-out2 | CAGTGATCAGCATCCACATGC | no | ** | 1,600 |
|  | Pax3prom39 | CTGGAGCCTGTGGACTTGGAT |  |  |  |
|  |  |  |  |  |  |
| * transgene insertion genotyping | | |  |  |  |
| ** replaces deletion | |  |  |  |  |
|  |  |  |  |  |  |
